# Subclinical Partial Attritional Rupture of the Flexor Digitorum Profundus as an Etiology of Atraumatic Trigger Finger

**DOI:** 10.1155/2017/8769369

**Published:** 2017-07-12

**Authors:** D. Anthony Bastian, Nicholas Kusnezov, John C. Dunn, Justin S. Mitchell, Miguel Pirela-Cruz

**Affiliations:** Department of Orthopaedic Surgery, Texas Tech University Health Sciences Center, William Beaumont Army Medical Center, El Paso, TX, USA

## Abstract

**Background:**

Trigger finger is a relatively common clinical entity. The etiology is most often attributable to stenosing tenosynovitis though traumatic cases have been described. When trigger finger is associated with an underlying flexor tendon rupture, previous cases have reported a clear association with overt laceration or previous trauma.

**Methods:**

We present the case of a 23-year-old male active duty military service member who presented with a characteristic history and clinical exam consistent with trigger finger. The symptomatic onset was gradual, with no history of inciting trauma.

**Results:**

Given symptomatic persistent triggering following failure of conservative management to include cortisone injections, the patient was taken for open A1 pulley release. Intraoperatively, the triggering was found to be attributable to a partial attritional rupture of the small finger flexor digitorum profundus tendon. Tendon debridement, tubularization, and A1 and partial A2 pulley releases were performed with subsequent resolution of triggering.

**Conclusion:**

We present the rare case of subclinical atraumatic attritional rupture of the FDP tendon to the small finger as a cause of clinically apparent trigger finger. This is an important consideration as the hand surgeon must be prepared to address more atypical pathologies.

## 1. Introduction

Trigger finger is a common condition typically arising spontaneously, with a reported lifetime prevalence of approximately 2.5 percent [1, 2]. Trauma is a known etiology of trigger finger and several risk factors have been implicated as contributing factors including diabetes mellitus and inflammatory arthropathy [2]. The pathology most often arises from nodularity of the flexor tendons, stenosis of the pulley system, or a combination, resulting in pain with catching and locking when the digit is brought from flexion to extension [2, 3]. Here, we present a unique case of atraumatic trigger finger which was taken to the operating room for A1 pulley release at which time it was discovered intraoperatively to be secondary to a subclinical partial attritional rupture of the small finger flexor digitorum profundus tendon.

## 2. Case Presentation

A 23-year-old right-hand dominant male active duty military service member was initially seen in our orthopaedic hand clinic upon referral by his primary care physician for further evaluation and management of a suspected trigger finger. The patient complained of six months of progressively symptomatic locking of the dominant small and ring fingers with pain over the palmar aspects of the bases of both digits. The patient was unable to recall any inciting trauma. His daily activities included repetitive manual activities involved with military service.

Physical exam was significant for painful active triggering of the small finger when brought from a position of maximal flexion to extension as well as palpable nodularity and tenderness to palpation over the palmar aspects of the small and ring finger metacarpophalangeal (MCP) joints. There was otherwise no extensor lag, and the patient was able to make a full composite fist.

Given the patient's history and clinical presentation, a cortisone injection was administered to the small finger A1 pulley and flexor tendon sheath. He reported complete pain relief following the injection and was sent to occupational therapy. The patient returned to orthopaedic hand clinic with continued pain relief but persistent triggering of the small finger six months later. With the exception of no tenderness, the exam was otherwise unchanged. Given that his symptoms were refractory to conservative management, he was taken to the operating room for small finger A1 pulley release.

**Figure 1:**
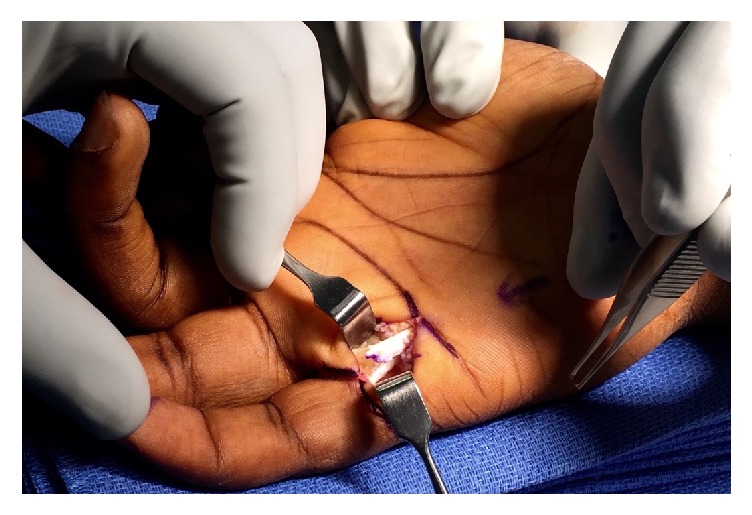
Intraoperatively, the triggering was found to be attributable to a partial attritional rupture of the small finger flexor digitorum profundus tendon.

**Figure 2:**
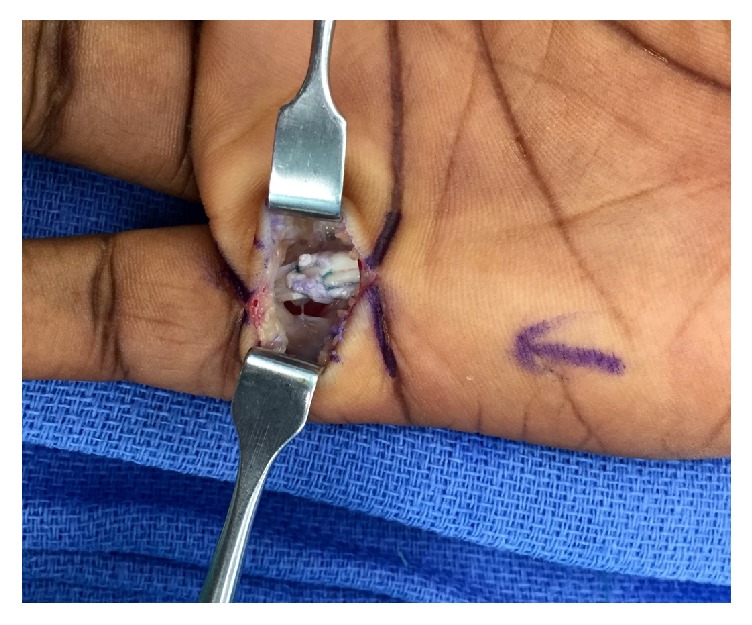
Given the extent of tendon disruption, the FDP was repaired and tubularized with 3-0 Ethibond suture.

A standard longitudinal incision was made overlying palmar aspect of the small finger MCP joint from the distal palmar crease to the palmodigital crease distally. The pulley was exposed and upon release a frayed, degenerative-appearing flexor tendon stump emerged (Figure 1). Interestingly, the patient displayed an anatomic variant as he did not have a flexor digitorum superficialis tendon to the small finger, relying solely on flexor digitorum profundus (FDP). The A1 pulley was completely released, exposing a chronic partial attritional rupture of the flexor digitorum profundus comprising the palmar 70% of the tendon. Given the extent of tendon disruption, the FDP was repaired and tubularized with 3-0 Ethibond suture (Figure 2). Taking the finger through a full range of motion, the repaired portion continued to engage on the proximal aspect of the A2 pulley; therefore, the proximal 50% of the A2 pulley was subsequently released. At the completion of the procedure, the patient was taken through full range of motion with no appreciable triggering and/or bowstringing of the flexor tendon.

Postoperatively, the patient was immobilized in a dorsal blocking splint for soft tissue rest for 1 week and subsequently enrolled in early active range of motion with occupational therapy. Follow-up 3 months postoperatively revealed excellent strength and range of motion with no further triggering.

## 3. Discussion

We present a unique case of an atraumatic partial rupture of the small finger FDP with a classic clinical presentation of trigger finger necessitating flexor tendon repair and A1 pulley release. This has previously not been reported in literature. Literature is replete with several case reports of trigger finger attributable to flexor tendon or pulley rupture following steroid injection or from laceration to the flexor tendon [1, 3–5]. Kimura et al. describe a patient who presented with subcutaneous flexor tendon bowstringing tendon about the thumb interphalangeal joint following the third dexamethasone sodium phosphate injection for trigger thumb [5]. The authors suggest that overutilization of corticosteroid injections can weaken the A1 pulley system and lead to eventual attritional rupture. Similarly, Nanno et al. presented a case of flexor tendon rupture of the thumb following corticosteroid injection, but in this case, the pulley ligaments were unaffected [3]. The patient in this case underwent two triamcinolone injections prior to A1 pulley release to relieve persistent tenderness and pain. Intraoperative evaluation revealed a partially ruptured flexor pollicis longus tendon though it did not require repair. In a similar case, Yamada et al. describe a patient with trigger finger, who, after treatment with triamcinolone, experienced uncomfortable snapping during normal use. Fujiwara and Ceran describe patients who developed flexor tendon rupture on trigger finger following laceration [1, 4]. While these cases present a possible etiology for our case, our patient improved clinically following injection and demonstrated no change in physical exam in the months following injection. It is more likely that the patient had experienced a subclinical trauma with a partial attritional rupture of the FDP to the small finger prior to his index visit to orthopedics. The degenerative nature of his partial tendon rupture did not present differently than the classic progression of trigger finger. In the other aforementioned cases, clinical presentation was strongly suggestive of rupture given acute bowstringing, painful swelling with loss of flexion, or a traumatic mechanism of injury [1, 3–6].

Both open and percutaneous techniques for A1 pulley release have been described [7]. Traditionally, open surgical approaches for A1 pulley release have consisted of either a perceivably more cosmetic transverse or potentially extensile longitudinal incision [7, 8]. Open approaches provide for direct visualization and protection of the neurovascular bundle as well as visualization of the completeness of the release. Conversely, while percutaneous A1 pulley release is minimally invasive, incomplete release has been described and persistent pain is a common finding in as many as 50% of patients. Our case supports the use of an open approach through a longitudinal incision should it need to be extended to allow for adequate tendon exposure and repair. The implications of this require the surgeon to be prepared for a tendon repair in a routine A1 pulley release.

We present the rare case of subclinical atraumatic attritional rupture of the FDP tendon to the small finger as a cause of clinically apparent trigger finger. This is an important consideration as the hand surgeon must be prepared to address more atypical pathologies.
